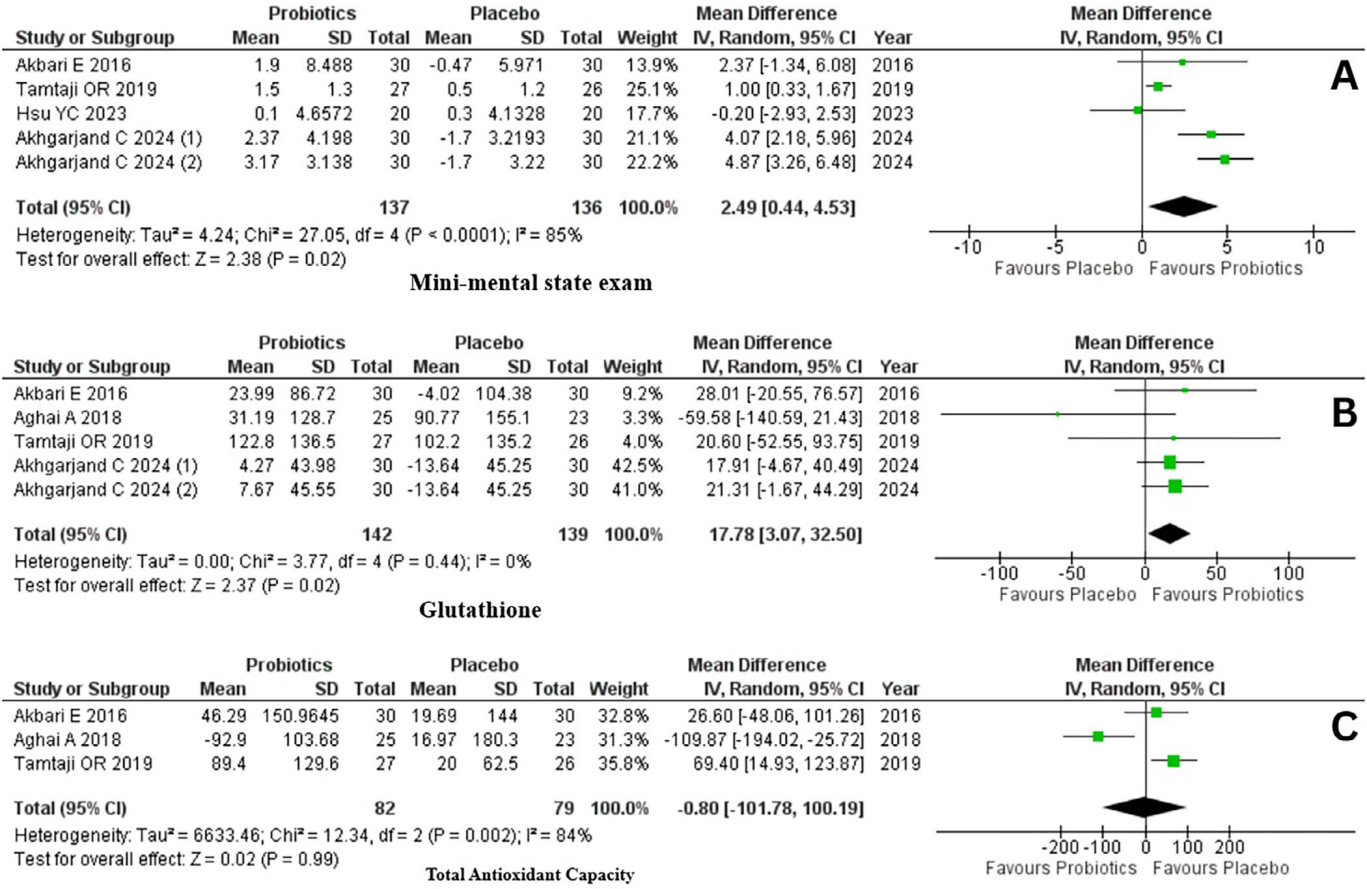# Effects of Probiotics on Oxidative, Cognitive and Metabolic Outcomes in Alzheimer's Dementia: A Systematic Review and Meta‐analysis of Randomized Controlled Trials

**DOI:** 10.1002/alz70857_102889

**Published:** 2025-12-25

**Authors:** Abdul Moeed, Hassan Waseem, Abdul Hannan Siddiqui, FNU Maria, Saba Yaqoob, Zulfiqar Haider Jogezai, Muhammad Moosa, Asma Saif, Muhammad Bilal, Fizza Batool, Areeba Ehsan, Muhammad Shoaib

**Affiliations:** ^1^ Dow Medical College, Dow University of Health Sciences, Karachi, Karachi, Sindh, Pakistan; ^2^ Allama Iqbal Medical College, Lahore, Punjab, Pakistan; ^3^ Shaheed Mohtarma Benazir Bhutto Medical College Lyari, Karachi, Sindh, Pakistan; ^4^ Aga Khan University, Karachi, Sindh, Pakistan

## Abstract

**Background:**

Alzheimer's disease (AD), being a deteriorative and progressive neurocognitive condition, constitutes nearly 70% of all the cases related to dementia and has a prevalence of 50 million globally. This study aims to analyze the probiotic's effect on neurocognitive function and markers of inflammation and oxidative stress in AD patients.

**Method:**

An exhaustive literature search was conducted across databases, including PubMed, Cochrane Central, and Google Scholar, for the relevant studies from inception to January 2025. Data from the included studies were extracted for relevant outcomes of interest. We used Review Manager version 5.4 to pool the mean differences (MD) and 95 % CIs for continuous outcomes. The random effects model was employed for statistical analysis, and the source of heterogeneity in the included studies was investigated through a sensitivity analysis.

**Result:**

Five randomized clinical trials, including 219 AD individuals, were added to this quantitative analysis. The results depict that the Mini‐Mental status examination score was significantly higher in the probiotics group (MD = 2.49, 95% CI: 0.44,4.53; *p* =  0.02; I2 = 85%). Probiotics also resulted in a higher level of glutathione (MD = 17.78 µmol/L, 95% CI: [3.07 to 32.50]; *p* = 0.02; I2=0%) and a lower malondialdehyde (MD = ‐1.54 µmol/L, 95% CI = ‐2.31 to ‐0.78; *p* <0.0001; I2 = 95) compared to placebo. Other outcomes were comparable between the two groups.

**Conclusion:**

The results show that probiotics effectively improve neurocognitive function in AD patients. Probiotics also increase antioxidants like glutathione and decrease inflammatory markers like malondialdehyde, leading to favorable outcomes.